# Hunting Bioactive Molecules from the *Agave* Genus: An Update on Extraction and Biological Potential

**DOI:** 10.3390/molecules26226789

**Published:** 2021-11-10

**Authors:** Misael Bermúdez-Bazán, Gustavo Adolfo Castillo-Herrera, Judith Esmeralda Urias-Silvas, Antonio Escobedo-Reyes, Mirna Estarrón-Espinosa

**Affiliations:** 1 Centro de Investigación y Asistencia en Tecnología y Diseño del Estado de Jalisco, A.C., Unidad de Tecnología Alimentaria, Camino Arenero 1227, El Bajío, Zapopan 45019, Mexico; mibermudez_al@ciatej.edu.mx (M.B.-B.); gcastillo@ciatej.mx (G.A.C.-H.); jurias@ciatej.mx (J.E.U.-S.); 2 Centro de Investigación y Asistencia en Tecnología y Diseño del Estado de Jalisco, A.C., Unidad de Servicios Analíticos y Metrológicos, Av. Normalistas No. 800, Guadalajara 44270, Mexico; aescobedo@ciatej.mx

**Keywords:** secondary metabolites, bioactive compounds, agave agro-wastes, biological activity

## Abstract

Agaves are plants used in the production of alcoholic beverages and fibers. Ever since ancient times, pre-Hispanic cultures in Mexico have used them in traditional medicine to cure different ailments. Over the years, studies of the active principles responsible for the therapeutic benefits of agaves have increased. Leaves and fibers are the main agro-wastes generated in tequila and mezcal production, while fibers are the main waste product in the textile sector. Different investigations have referred to the agro-waste from agave processing as a source of bioactive molecules called secondary metabolites (SM). Among them, phenols, flavonoids, phytosterols, and saponins have been extracted, identified, and isolated from these plants. The role of these molecules in pest control and the prospect of metabolites with the biological potential to develop novel drugs for chronic and acute diseases represent new opportunities to add value to these agro-wastes. This review aims to update the biological activities and recent applications of the secondary metabolites of the genus *Agave*.

## 1. Introduction

The agaves are perennial plants that use Crassulacean Acid Metabolism (CAM), which allows their adaptation to arid weather ecosystems [1]. The *Agave* genus is the largest of nine subgenres of the *Asparagaceae* family, with 210 species, of which 119 are endemic in Mexico; thus, it is considered the center of diversity of these plants [2,3]. Geographically, it is distributed within an area stretching from the southern United States to Ecuador [2].

The production of alcoholic beverages such as tequila, mezcal, and bacanora represents the most traditional use of these plants [4]. Nevertheless, the high demand for these products has led to the production of different agro-wastes that contaminate with the potential of being dangerous for the environment. Between 2015 and 2019, 1.434 billion liters of tequila were produced, which created an estimated 5,168,200 tons of agave leaves in waste. While the mezcal industry reportedly produced 27,663,459 liters, no official data regarding agro-waste generation of this beverage exist [5].

In the textile industry, obtaining fibers from *Agave lechuguilla* Torr. leaves is an important economic activity in this sector in Mexico. Within Mexico, the states of San Luis Potosí, Coahuila, Nuevo León, Zacatecas, Durango, and Tamaulipas are the main producers of this raw material, from which only 15% is product and 85% is an agro-waste called “guishe”. This industry generated 150 tons of guishe per year [6,7,8]. Agro-waste production in the form of leaves and fibers has become an environmental concern. 

### Agave in Traditional Medicine

In ancient times, pre-Hispanic cultures in Mexico such as the Mayans and the Aztecs used agaves for medicinal purposes because of their therapeutic properties [9,10]. For example, the Mayans obtained leaf juice from the *A. fourcroydes* Lem. to heal wounds via topical application. In contrast, the roots and leaves of *A. angustifolia* Haw. were used to treat cutaneous and excretory skin infections and treat snakebite [11]. 

The Aztecs squeezed the fresh leaves of *A. atrovirens*, *A. americana*, and *A. potatorum* Zucc. to obtain concentrated sap. Later, they mixed it with hot urine, salt, honey, and some other medicinal plants. Other preparations involved *Comelina pallida* as a hemostatic agent. Finally, the mixture was applied to deep wounds [12].

The Tarahumara indigenous culture from Chihuahua domesticated a few *Agave* species and used these plants in medicinal preparations that consisted of applying leaf juice to festering sores. The sap was used to treat ocular infections, while other preparations with whole leaves were used to cure headaches [13]. 

Worldwide, different indigenous cultures have used these plants for their therapeutic effects. They have employed some species in the treatment of insect-borne diseases [14], rheumatic inflammatory affections [15], and nervous system diseases such as meningitis and sciatica [16]. Other medicinal preparations were preserved over the years, for example, the remedies prepared from *A. angustifolia* Haw., described by García Mendoza et al. [17]. This and other medicinal uses of different *Agave* species are summarized in Table 1.

Until now, the medicinal applications of agave plant constituents were the prelude to many research studies conducted to discover the bioactive compounds responsible for their biological activity by applying different extraction technologies and analytical tools.

This review aims to describe various recent studies on secondary metabolites that have been extracted, isolated, and identified in different *Agave* species. It also describes those studies that have examined the bioactive properties of specific molecules and the biological activities of crude extracts with potential applications.

## 2. Extraction Methods Used to Recover Polyphenolic Compounds from *Agave* Agro-Waste

A previous review by Almaraz et al. [24] described the phenolic compounds of agaves. This section updates the information on the extraction and identification of different polyphenolic compounds and the factors that influence their extraction and occurrence in the *Agave* genus.

Phenolic compounds are polar molecules that possess an aromatic benzene ring, substituted with one or more hydroxyl (-OH) groups while flavonoids have more than one phenyl ring. Their structure has a heterocyclic ring of benze-γ-pyrane, which is hydroxylated in distinct patterns [25]. Both types of metabolites can be methylated, glycosylated, and acylated. These structural modifications have been attributed to biochemical reactions of the vegetal metabolism and they impact the biological activity [26].

Because of the high polarity of glycosylated polyphenols, aqueous mixtures with a polar organic solvent have been employed to maximize their recovery. Barriada-Bernal et al. [27] used two extraction stages with 60% and 30% (*v*/*v*) ethanol, respectively, on *A. durangensis* Gentry flowers, and were able to identify through HPLC-UV-VIS, quercetin-3-*O*-[rhamnosyl-(1→6)-galactoside], kaempferol-3-*O*-[rhamnosyl-(1→6)-glycoside], kaempferol-3,7-*O*-diglycoside, and quercetin-3-*O*-glycoside as the most abundant molecules. Similarly, Almaraz-Abarca et al. [28] employed 60% (*v*/*v*) methanol on *A. victoriae-reginae*, *A. striata* Zucc., and *A. lechuguilla* Torr. leaves. They identified 25 glycosylated flavonoids and high levels of 3-*O*-glycosides of kaempferol were reported in these species. Besides, the presence of the glycosides of isorhamnetin, quercetin, and herbacetin were also reported. 

Morreeuw, Escobedo-Fregoso, et al. [29] investigated the effect of binary aqueous mixtures solvents of *A. lechuguilla* Torr. leaves, and found that an ethanol–water mixture of 70:30 (*v*/*v*) enhanced the recovered yields of cyanidin and delphinidin. Conversely, an aqueous methanol mixture 60:40 (*v*/*v*) resulted in a more suitable extract for flavonoids due to its high polarity, and it obtained the highest yields of isorhamnetin and hesperidin. 

Later, Morreeuw, Castillo-Quiroz, et al. [30] confirmed with HPLC-MS/MS that the hydroalcoholic mixture 70:30 (*v*/*v*) of *A. lechuguilla* Torr. was plentiful in mono-, di- and tri- glycosylated derivatives of apigenin, isorhamnetin, quercetin, and anthocyanins. Additionally, it was observed that the presence of more than one glycoside moiety was influenced by regional factors. Therefore, those extracts that belonged to drought regions accumulated -di or -tri glycosylated flavonoids; these compounds can provide better tolerance to drought stress [30]. Other studies on other *Agave* species demonstrated that drought stress induced an increase in these compounds and other secondary metabolites [31,32].

Morán-Velázquez et al. [33] investigated the use of accelerated solvent extraction as applied to young leaf spines of *A. fourcroydes* Lem. The extracts were plentiful in proanthocyanidins; this was confirmed by UHPLC-MS/MS. The accumulation of these metabolites into the fiber of young spines was possibly due to the laccase enzyme activity implied in the proanthocyanindins biosynthesis pathway. The authors associated this finding with the high abundance of (+)-catechin, (−)-epicatechin, and their glycosylated derivatives, as well as flavonoids which are the precursors of these compounds. Iser et al. [34] found the presence of tannins, phenols, and flavonoids in ethanolic and aqueous extracts from stems of this species.

In general, the variation in the total phenolic and flavonoid contents and other secondary metabolites has been described in relation to the type of *Agave* species, plant age, geographical origin, and solvent [35,36,37,38,39,40]. Ben Hamissa et al. [41] showed that high temperatures and pressures also improved the yield of phenols and flavonoids. Furthermore, emerging extraction processes such as ultrasound-assisted extraction also enhanced the yield of these compounds and reduced extraction times [42].

Pretreatments prior to the process and extraction type also influence the nature of the recovery of polyphenols from agaves. Contreras-Hernández et al. [43] found through HPLC-MS/MS that the phenolic composition of acetone extracts (85% *v*/*v*) from leaves of *A. durangensis* Gentry pretreated at a temperature of 120 °C and separately with an ultrasound had different phenolic profiles. Higher temperatures led to the partial degradation of lignin and the chemical conversion into new phenol molecules. Alternatively, the ultrasound-assisted extraction increased the content of specific flavonoids, such as quercetin, rutin, procyanidin B2, and others. Cavitation phenomena promote the rupture of the vacuoles, which leads to the release of aglycones from these compounds and consequently increases the yield of the recovered phenols [44].

A study reported by Avila-Gaxiola et al. [45] proved that a temperature of 100 °C applied in the pretreatment stage did not degrade the recovered phenolic acids of an aqueous extract of *A. tequilana* Weber. The thermal pretreatment promoted the generation of new phenolic compounds, such as 4-hydroxy-benzoic acid, pyrocatechol, acetovanillin, vanillin, and 2-furoic acid. 

Because agave extracts are susceptible to undesirable degradation reactions by biologic contamination or high temperatures involved in their pre-processing, the stability and bioactivity of polyphenols and other secondary metabolites might be compromised. To overcome this, Santana-Jiménez et al. [46] found that the irradiation by UV-C light at doses of 10.93 mJ/cm^2^ for 30 seconds inactivated the native microbiota from piña crude extracts of *A. tequilana* Weber and did not affect the antioxidant or extract color. In this way, the irradiation by UV-C light for short times may represent a suitable technology to preserve agave crude extracts.

Acid hydrolysis is another pretreatment used in the extraction of phenolic compounds. One study reported an increase in the yield of these compounds in hydrolyzed extracts of *A. lechuguilla* Torr. The extracts showed aglycones and dimeric flavonoids, such as quercetin-4β-afzelechin [47]. Contrary to these results, Mitchell et al. [48] reported trace amounts of phenolic acids in hydrolyzed leaf extracts of *A. americana*, *A. fourcroydes*, and *A. tequilana*, respectively.

## 3. Bioactivity of Identified Phytochemicals from the *Agave* Genus

This section focuses on the description of the biological activities of identified and isolated polar and non-polar compounds to show the potential of novel bioactive molecules that were found in the literature related to the *Agave* genus. This information is summarized in Table 2.

### 3.1. Polar Compounds: Phenols and Flavonoids

The biological potential of polyphenols from different *Agave* species has been studied and assessed in vivo. The hydroalcoholic extract of *A. lechuguilla* showed insecticide activity against *Bemicia tabaci* at LD_50_: 1057 mg/L after 72 h, no positive control was used [50]. Another study reported the insecticide activity of aqueous methanol extracts on *Sitophylus oryzae*, at LD_50_: 10.55 µg/insect after 72 h vs. malathion at LD_50_: 2.34 µg/insect after 24 h [49]. Interestingly, an *A. americana* L. extract exerted repellent activity at RC_50_: 0.055 µg/cm^2^ while malathion did not present this activity. Besides, the chemical profile of *A. americana* L. presented flavonoids such as quercetin, isorhamnetin, kaempferol, and their glycosylated derivates and an ellagic acid glycoside was also identified, with the repellent activity associated to these compounds [49].

The anti-inflammatory and immunomodulatory activity of leaf extracts from *A. desmettiana* Jacobi, *A. angustifolia* Haw. cv. *marginata*, *A. americana* L., *A. americana marginata* Trel, and *A. pygmaea* Gentry were studied by El-Hawary et al. [51]. In contrast, *A. pygmaea* Gentry and *A. angustifolia* Haw. cv. *marginata* exerted the highest immunomodulatory activity at a concentration of 200 mg/kg on in vivo inflammation models and decreased the concentration levels of pro-inflammatory cytokines, which were different and lower than indomethacin administration. Alternatively, the production of IL-10 cytokine increased, which promoted an anti-inflammatory effect. Overall, these species showed different phenolic acids, glycosylated flavonoids, and homoisoflavonoids in their metabolomic profile, such as fukiic acid, sinapic acid, psicidic acid, methyl gallate, rutin, kaempferol, quercetin glucosides, eucomol, and dyhydroeucomin, and its occurrence was associated with such effects.

Anticancer activity has also been seen against lung cancer cell-lines in ethanolic leaf extracts from *A. lechuguilla* Torr. The crude extracts showed 75.7% of cellular growth inhibition in SK-LU-1 cells at IC_50_ 50 µg/mL vs. the anticancer drug Paclitacxel used as a positive control with 1.2% at 5 µg/mL. However, fractionated extracts presented this effect at a minor concentration (IC_50_: 6.96 µg/mL) due to the elimination of residual sugars from extracts that are the substrates of these cell lines. These findings indicated that residual sugars in crude extracts attenuated the biological activity [47].

Both antifungal and antidiabetic properties were reportedly seen in *A. americana* L. leaf extracts. Two studies found that the bioactive potential is selective and depends on the nature of the isolated molecule. Puerarin and apigenin isolated from this species showed greater inhibitory effects against *A. oryzae* α-amylase, while p-coumaric acid proved a major inhibitor to human α-amylase at IC_50_: 98.8 µM, with an inhibition rate 2.3 times higher than acarbose. Thus, isolated flavonoids may be used as therapeutic agents for postprandial glycemia [52,53].

**Figure 1:**
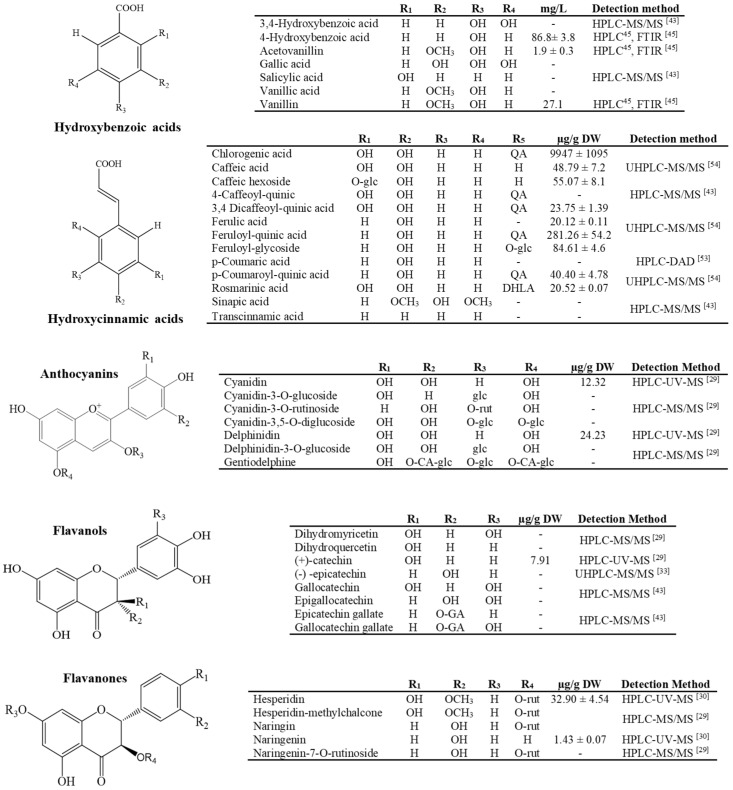
Current phenolic acids and flavonoids identified and quantified in the *Agave* genus, CA = cafeic acid, GA = gallic acid, rut = rutinoside, glc = glucoside, QA: quinic acid, DHLA= 3,4-dihydroxiphenillactic acid [29,30,33,43,52,53,54].

**Figure 2:**
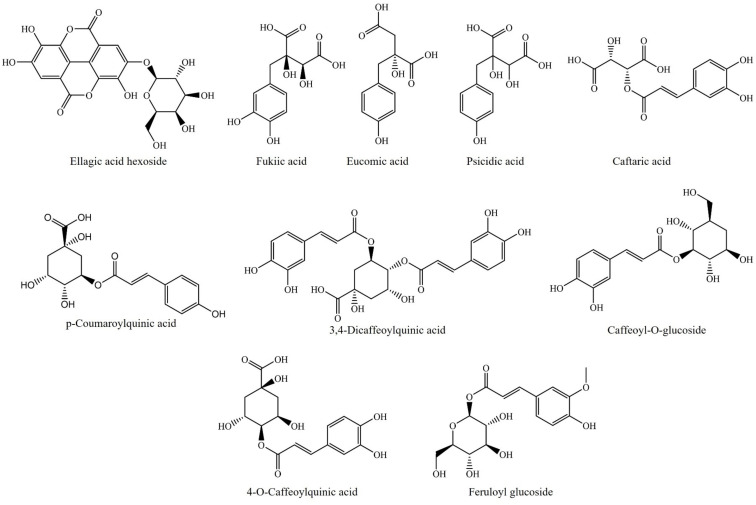
Current phenolic acids identified in the *Agave* genus.

**Figure 3:**
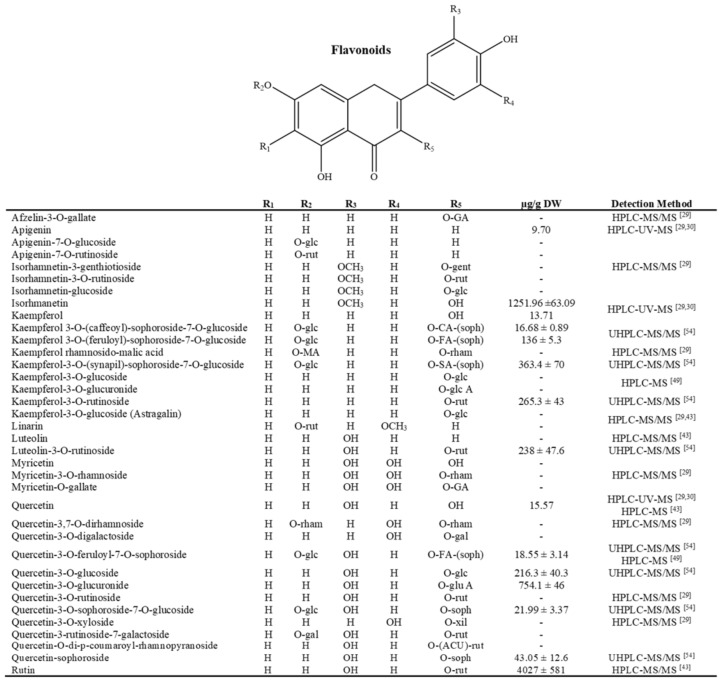
Current flavonoids identified and quantified in the Agave genus, glc = glucose; rut: rutinoside; gal = galactoside; rham = rhamnoside; soph = sophoroside; glu A = glucuronic acid; xil: xyloside; GA = gallic acid; gent = gentrobioside; SA = sinapic acid; FA = ferulic acid, ACU = cumaric acid; MA= mallic acid [29,30,43,49,54].

Contreras-Hernández et al. [43] confirmed that the presence of phenolic compounds that were identified in *A. duragensis* Gentry leaves enabled the production of industrial enzymes during fermentation with *A. oryzae* and *N. crassa*. Another study of this species reported that fibers worked as a growth medium for microgreens because of the presence of phenolic acids and glycosylated flavonoids, which adopted a protector role during their development [54]. Some of these compounds include the glycosides and acyl glycosides of kaempferol and quercetin and novel phenolic acids (illustrated in Figure 1, Figure 2 and Figure 3). 

### 3.2. Non-Polar Compounds: Saponins

Saponins are a class of metabolites with both hydrophobic and hydrophilic properties. Their versatility as bioactive agents has been described through various bio-guided studies of the *Agave* species. From these plants, nearly 28 steroidal saponins and 86 glycosylated saponins have been isolated; however, these numbers are still increasing. These compounds have shown antifungal, anti-inflammatory, cytotoxic, anticancer, and hemolytic activities [65].

Santos-Zea et al. [55] investigated anticancer activity against pulmonary cancer cell-lines by applying enriched fractions of tetra and penta-glycosylated steroidal saponin from *A. salmiana* sap. The author found that the fraction that showed agavoside C and gentrogenin pentaglycoside contributed to cell death by necrosis at IC_50_: 108.4 ± 11.5 µg/mL, while the fraction that presented magueyosides B, C, and kammogenin tetraglycoside induced apoptosis at IC_50_: 82.7 ± 3.6 µg/mL. Such effects were similar to the anticancer drug cisplatin which exerted necrosis and apoptosis at 10 µg/mL. Therefore, the anticancer activity of saponins depended on the glycosylated saponin derivative.

Furthermore, different fermented fractions of *A. sisalana* sap have been reported to exert anticancer activity but selectively through their different metabolic profiles. A fermented fraction from *A. globiformis* inhibited the growth of Caco-2 cell lines, while a *Gordonia* sp. fraction reduced Hep-G2 viability. Moreover, magueyoside B, and kammogenin glycoside were the most abundant in this extract and were associated with this bioactivity [57]. Another study showed that steroidal saponin, isolated from *A. marmorata* Roezl inhibited the NF-κB activation which modulates the inflammatory pathway of different diseases at IC_50_ of 0.01 µg/mL in RAW 264.7 macrophages [58].

The modulation of the neuroinflammatory response in rats was investigated by Herrera-Ruiz et al. [56] and it was reported that the leaf extracts of *A. tequilana* Weber and *A. americana* L. *marginata* Hort. at a dose of 125 mg/kg diminished TNF-α and IL-6 concentration levels compared to indomethacin at a dose of 5 mg/mL. In this research, cantala-saponin-1 was also isolated from an *A. americana* L. *marginata* Hort extract and it similarly decreased the concentration levels of these cytokines at 5 and 10 mg/kg of indomethacin. Alternatively, the administration of the extracts and the isolated saponin stimulated the production of the anti-inflammatory cytokine IL-10 [56]. Another study stated that the hydrolysate juice extract of *A. sisalana* Perrine did not exert mutagenic, cytotoxic, and neoplastic effects on Vero cells at a concentration of 50 mg/kg. At the same time, the positive control of cyclophosphamide showed clastogenic potential at the same dose [60]. Moreover, the hydrolysate extract reduced the levels of reactive oxygen species at 50 µg/mL. Interestingly, the author confirmed through HPLC-UV-vis a mixture of saponins with a variety of sugar moieties in hydrolysate extract [60]. Therefore, saponins could be safe and novel anti-neoplastic drugs compared to conventional chemotherapy agents.

Steroidal saponins have also showed gastroprotective activity. A recent study proved that isolated glycosylated steroidal saponin from *A. angustifolia marginata* did not produce hemolytic alterations in gastric tissue by the presence of different glycoside chain substitutions. This structural feature also reduced the affinity of cholesterol to the erythrocytes membrane and gave a cytoprotecting effect in these cells and non-cytotoxic damage [61]. Another study assessed the effect of a methanolic extract and a saponins-enriched fraction of *A. seemanniana* Jacobi leaves; both reduced ulcer severity in a similar way to ranitidine at 50 mg/kg. However, their effectiveness was at a concentration of 200 mg/kg [62]. The extracts also displayed anti-inflammatory and analgesic effects at a dose of 100 mg/kg. At this concentration, the therapeutic action was similar to aspirin (100 mg/kg) and indomethacin (20 mg/kg). In addition, the author confirmed through NMR the presence of glycosylated steroidal saponins in the active fractions [62]. 

The healing properties against ulcerative injuries in the rat colons were also investigated by applying extracts of *A. americana* Linn leaves. The effective dose of the extract was 200–400 mg/kg, and it reduced the catalytic activity of myeloperoxidase and lipoperoxidase, which are enzymatic markers involved in the inflammatory response of intestinal ulcerative colitis. After the application, the regeneration of the epithelial cells of the mucosa was observed. These healing effects were reached on the 11th day of application at a concentration of 400 mg/kg [66]. It is worth mentioning that at this concentration the extracts decreased the inflammation parameters such as colon length and weight more than acetic acid (4%) and indomethacin (7.5 mg/kg), which were used as positive controls in both studies. Besides, *A. americana* Linn leaves extracts had similar action to the standard drugs prednisolone and 1,3,3,3 tetra-ethoxy-propane used to treat this illness [66,67]. 

Steroidal glycosylated saponins were also identified in leaf extracts of *A. americana* var. *marginata*, *A. americana* L., *A. angustifolia* Haw cv. *marginata*, *A. desmettiana* Jacobi, and *A. pygmaea* Gentry, and the anti-inflammatory and ulceroprotective activities were attributed to these compounds, as well as fatty acid amides [51].

The study of the gastroprotective effect of isolated hecogenin of *A. sisalana* Perrine leaf was described on two ulcerative models, the ulcerative injuries were induced by ethanol and indomethacin. Santos Cerqueira et al. [68] found that a dose of 90 mg/kg p.o. decreased the percentage of ulcerative injuries on the rats’ stomachs, similar to commercial drugs such as ranitidine (100 mg/kg), *N*-acetylcysteine (300 mg/kg), and misoprostol in both ulcerative models. According to these findings, the author stated that hecogenin involved an antioxidant protector mechanism which consisted of the protection of thiol groups of non-protein molecules of gastric mucosa, antisecretory acid action, module inflammation, and through the regulated stimulation of prostaglandins biosynthesis. Additionally, the effective dose of hecogenin inhibited lipid peroxidation and myeloperoxidase release from neutrophils in the injury sites, which prevented the propagation of reactive oxygen species [68].

Overall, the obtention of natural products from agave agro-wastes might be advantageous in designing new therapies to treat gastrointestinal inflammatory diseases with novel antiulcerogenic agents.

Regarding the recovery of saponins, the assisted-ultrasound extraction was used on the bagasse of *A. salmiana*. The conditions that increased yields were 400-W, 24 kHz, and 60 °C. It is not recommended to use hydroalcoholic mixtures instead of water due to the low propagation of the ultrasound in those solvent systems [69]. Another technology employed on this agro-waste was supercritical fluid extraction assisted by an ultrasound. Increased antioxidant activity in the extracts was observed and the hexaglycosylated derivatives of hecogenin were identified among other steroidal saponins [70].

### 3.3. Phytosterols 

Phytosterols are hydrophobic compounds, and their base structure is cholesterol. In plants, these metabolites do not occur in free form; rather, they can be presented as conjugates of esters, glycosides, and acyl groups [71].

**Figure 4:**
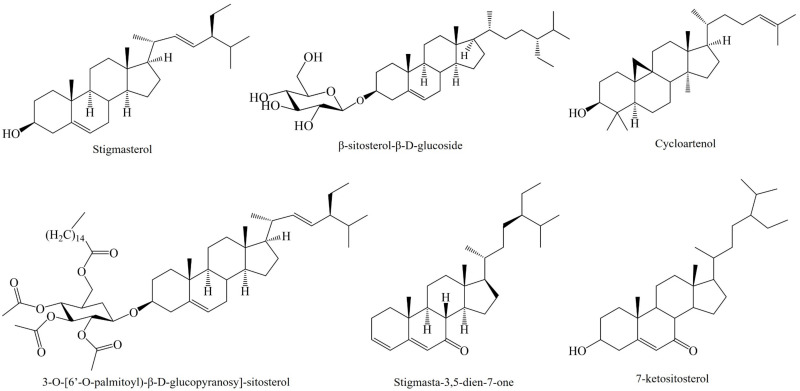
Current phytosterols identified and isolated in the *Agave* genus.

A study using ultrasound-assisted extraction in the piña of *A. angustifolia* Haw. had high extraction yields of β-sitosterol-d-glycoside in short periods of the process compared to maceration extraction [72]. Hernández-Valle et al. [64] investigated the anti-inflammatory effect of acetone fractions of this species on a mice ear edema inflammation model induced by TPA. According to the authors, AaF16 was the most active fraction and decreased the levels of IL-1β, IL-6, and TNF-α at 0.8 mg/ear; this effect was greater than that of dexamethasone (0.1 mg/ear). Besides, the topic application of the fraction at this concentration showed minor mononuclear inflammatory cell infiltration than dexamethasone and TPA on skin and cartilage. The chemical profile obtained by NMR of the active fraction confirmed the presence of different phytosterols such as β-sitosteryl glycoside, stigmasterol, and a peracetylated derivative of 3-*O*-[6′-*O*-palmitoyl)-β-d-glucopyranosyl-sitosterol] [64], which are depicted in Figure 4.

Gutiérrez Nava et al. [63] compared the effect of the acetone extract of *A. tequilana* Weber leaf on rats with systemic erythematosus lupus (SLE) against methotrexate and prednisone, which are immunosuppressant and anti-inflammatory drugs used to treat this illness. The authors found that methotrexate (3 mg/kg) diminished the titer of DNA autoantibodies, but increased proteinuria levels; thus, it had a nephrotoxic effect. Conversely, *A. tequilana* Weber extracts reduced proteinuria associated with SLE, and decreased the titer of DNA autoantibodies, but not significantly. On the other hand, both drugs dropped the levels of IL-1β, IL-6, TNF-α, and INF-γ which are similar to the acetone fraction of *A. tequilana* Weber (50 mg/kg) which also stimulated the production of the IL-10 cytokine, a key cytokine that regulates the autoimmune response [63]. Through GC-MS, phytosterols and terpenes such as β-sitosterol-glycoside (0.1995), stigmasta-3,5-dien-7-one (0.0285), cycloartenol (0.019), and a phytol (0.1615) mg/g extract, were identified and quantified which were associated with this activity [63].

Furthermore, in the piña extracts from *A. angustifolia* Haw., *A. cupreata* Trel. & A. Berger, *A. salmiana* ssp. *Crassispina*, *A. salimiana* var. *salmiana*, *A. karwinskii* Zucc., leaf extracts of *A. atrovirens*, *A. decipiens* Baker, and a lipophilic fraction of fiber of *A. sisalana*, 7-ketositosterol, stigmasta-3,5-dien-7-one, stigmasta-4-en-3-one, stigmasta-4-en-3,6-dione and stigmastane-3,6-dione were found. However, no information was reported in this research about their biological activity [73,74,75,76].

## 4. The Role of *Agave* Extracts as Biopesticides

The application of agave crude extracts from agro-wastes has been on a diversity of applications on livestock and agricultural sectors. Since they are useful for pest insect management, larvae and intermedial organisms are considered vectors of parasitic diseases and fungi crop infestations.

### 4.1. Insecticide Activity

Lopes et al. [77] appraised the use of no contaminant solvents and applied a hydroalcoholic extract from *A. sisalana* Perrine leaf against adult females of *Dactylopius opuntiae*. The median lethal dose (LD_50_) was 43 mg/mL after 10 h of application. Alternatively, the combination between extract and synthetic insecticide in their LD_50_ enhanced the mortality percentage because of the synergy between the insecticide compounds and extracts. This may be a strategy to introduce agave plants as biopesticides and diminish high concentrations of synthetic products in fields.

Guimarães de Oliveira et al. [78] observed that *A. sisalana* Perrine leaf juice had a cytotoxic effect on the hemocytes cells of *A. aegyptii* and increased at a dose of 6 mg/mL. As a result of the application of the extract, it incremented nitric oxide production, which stated a defense response against the secondary metabolites of the extract. On this agro-waste, saponins, triterpenoids, alkaloids, tannins, and flavonoids were detected by qualitative analysis and might be associated with this effect [79]. 

The leaf of *A. sisalana* Perrine has also been used for the biologic control of *Callosobruchus maculatus*. A concentration of 100 mg/mL reduced the oviposition, larvae survival and pupae stage, and their population on bean infested seeds [80].

The application of the leaf extract of *A. americana* var. *marginata* Trel. had a 70% mortality rate against *Brevicoryne brassicae* at a concentration of 0.75 mg/mL after 3 h of application [81]. Alternatively, this species activity showed 70.3% and 92% of mortality against *Aphis gossypii* at 25 mg/mL after 24 and 36 h of application, respectively. However, the extract was toxic for *Artemisia salina*, a non-target organism [82].

Cunha Pereira et al. [83] identified β-caryophyllene in the aqueous extract of *A. americana* L. leaves as the most abundant compound, which had no deleterious effects in two species of bees; even at a high extract concentration, it showed CL_50_ of 127.4 and 122.2 ng/µL for *A. mellifera* y *P. helleri*, respectively. In contrast, imidacloprid had CL_50_ of 0.09 and 0.11 ng/µL in both species; therefore, the application of this pesticide resulted in more noxious effects than the extract. Moreover, Marafeli et al. [84] sprayed an aqueous extract of this species on coffee leaves infested with *Olygonychus ilicis* and it presented a 100% mortality rate at 4% (*v*/*v*) after 72 h of application. 

Other species, such as *A. angustifolia* Haw., *A. tequilana* Weber, *A. sisalana* Perrine, and *A. attenuata* showed insecticide activity against *Bemicia tabaci*, *Tetranychus urticae*, and *Diuraphis noxia* [85,86,87]. Particularly, the extract of *A.attenuata* presented methyl-jasmonate, 2,5-dimethyl-3,4-hexanediol, and n-hexadecanoic acid as the most abundant metabolites, which might effect insecticide activity [88].

### 4.2. Molluscicide Activity

The agave crude extracts have been used for the biological control of mollusk. These organisms are vectors of diseases that affect human health due to are host of dangerous parasites. 

From the above, different research has found that some *Agave* species such as *A. filifera*, *A. celsii*, *A. sisalana*, *A. decipiens* Baker, and *A. lophanta* exerted molluscicide activity against *Biomphalaria alexandrina* [75,89,90,91], a snail vector of *Schistosoma mansoni*, which causes schistosomiasis. The effective lethal dose and extract type are summarized in Table 3. 

Another study by Bakry and Salwa A.H [90] found that a dose of 22 mg/L of the methanolic leaf extract of *A. celsii* reduced the activity of the glycolytic enzymes of *B. alexandrina* and led the snails to an anoxia state. Alternatively, the extract administration caused the release of cellular content associated with the low enzyme activity of their energetic metabolism and diminished the oviposition rate, which prevented the propagation and infectivity of *S. mansoni* to *B. alexandrina.*

More studies have shown that the *A. legrelliana* Jacobi leaf juice extract exhibited molluscicide activity against *Praticolella griseola* with an 82.5% mortality rate after seven days [93]. Besides, this agro-waste was able to diminish notably the cardiac frequency of *Fossarium cubensis* within a range of 0.105 to 7.718 mL/L after three days of application [107]. 

Molluscicide activity of *A. americana* var. *expansa* was also assessed; the aqueous extract caused 65% of mortality against *Melanoides tuberculata* at LD_50_ of 0.86 mL/L after 24 h of exposition [94]. However, another study employed a butanol extract, and it showed an ovicide effect against *Indoplanorbis exutsus, Lymnaea luteola, and Gyraulus convexiusculus* at LD_50_ of 21.95, 18.56, and 16 mg/L, respectively [95].

The molluscicide activity of aqueous, ethanolic, and butanol extracts of *A. sisalana* Perrine leaf against *Pomacea canaliculate* was appraised. Particularly, aqueous extracts showed the highest molluscicide effect at LD_50_ of 35.3 mg/L [96]. Besides, the effective concentration decreased superoxide dismutase activity due to the oxidative stress produced by the phytochemicals of the extract. Similarly, Hamed et al. [97] observed a modification in the activity of the antioxidant enzymes within the effective molluscicide concentration range. Thus, it might have involved a defense response brought about by secondary metabolites. 

The molluscicidal activity of saponins was also investigated. A study showed that isolated tigogenin diglycoside from *A. angustifolia* leaves killed 90% of the *B. alexandrina* population at a concentration of 61.4 mg/L after 24 h of exposition [108]. Furthermore, in other research, steroidal glycosylated saponins from *A. franzosinii* P. Sewell and *A. angustifolia* exhibited this effect at LD_50_ similarly to niclosamide and praziquantel, which are synthetic molluscicides [109]. Moreover, neoruscogenin and neohecogenin isolated from *A. decipiens* Baker leaves had 90% mortality against this snail species at concentrations of 13 mg/mL and 6 mg/mL, respectively, after 24 h of application [75]. Considering this evidence, saponins might be an alternative to replace synthetic pesticides that might be noxious to the environment.

### 4.3. Larvicide Activity

Concerning larvicide activity, Kajla et al. [106] described that thermal stress on *A. angustifolia* var. *marginata* leaves swelled the concentrations of secondary metabolites in the extracts; this enhanced the mortality index against larvae *Aedes aegypti* at LD_50_ of 28.26 µg/mL after 12 h of application. Alternatively, the reduction in LD_50_ to 19.15 µg/mL was achieved, but during a longer exposition time (24 h). On the other hand, the extract also showed larvicide activity against *Culex quinquefasciatus* and *Anopheles stephensi*, but at 100 µg/mL.

Another study stated that the application of *A. sisalana* Perrine leaves juice killed *A. aegypti* larvae at LD_50_ of 4.5 mg/mL; the author reported that the high concentration of this agro-waste exhibited hemocytes necrosis and reduced nitric oxide concentration, which is involved in the immune response of this organism, after 24 h of application [98].

Furthermore, leaf juice extracts from *A. sisalana* Perrine obtained with water and other organic solvents were appraised. The aqueous extract killed *C. quinquefasciatus*, *A. aegypti*, and *A. stephensi* larvae at LD_50_ of 86, 76, and 75 mg/L, respectively. The highest mortality rate was on the III/IV growth stage of larvae. In contrast, the methanolic extract showed greater mortality at an LD_50_ of 36 mg/mL [99]; however, applying an organic solvent in plant molluscicides might represent an environmental risk. Moreover, Pizarro et al. [100] assessed the larvicidal effect of aqueous extracts of this species against *C. quinquefasciatus*, and it showed a mortality of larvae at LD_50_ of 322 mg/mL. In this research, the fractionation of crude extracts, was performed which enhanced the effect and reduced the LD_50_ to 204 mg/L, due to the increase in the saponins content and the elimination of undesirable compounds.

The larvicidal activity of *A. cantala* (Haw.) Roxb. Ex Salm-Dyck against *Plutella Xylostella* (L.) was weak at a 60 mg/L concentration with 35.77% mortality. However, a 25 mg/mL concentration prolonged the growth periods of the pupae and larvae, decreasing the fecundity rate of adult females and the hatching of the egg by 94.5% [101]. Thus, the disruption of reproductive cycles represents a strategy for plague control of other organisms that affect industrial crops.

### 4.4. Nematicide Activity

The field of bionematicides development through agave plants has gradually broadened as different research has proved the efficacy of agro-waste against different phytonematodes. Fábio et al. [102] observed that fresh leaf juice of *A. sisalana* Perrine exhibited 99.2% of mortality against *Radopholus similis* after 48 h, at 25% (*v*/*v*). In contrast, fermented juice at that concentration caused a phytotoxic effect on banana plants. Similarly, Damasceno et al. [103] found that the fresh juice of *A. sisalana* Perrine displayed 100% mortality on infested tomato crops with *Meloidogyne javanica* at 20% (*v*/*v*). In addition, the agro-waste reduced nematode egg masses and the number of galls per plant. Conversely, fermented juice caused the inhibition of beneficial soil organisms. Likewise, Dos Santos Fernandes et al. [104] confirmed the nematicide activity of the liquid agro-waste of *A. sisalana* Perrine (20% *v*/*v*) against *Scutellomena bradys* with a 100% mortality rate after 24 h.

Another approach on the bionematicides from agave is the management of gastrointestinal nematodes in the livestock sector. Different *Agave* species such as *A. angustifolia* Haw., *A. americana* L., *A. tequilana* Weber and *A. sisalana* Perrine exhibited nematicide activity against *Panagrellus redivivus, Meloidogyne incognita, Haemonchus* spp., *Oesophagostomum* and *Trichostronglyus*. Those studies used methanolic and aqueous extracts, which killed nematodes within 24 to 72 h of application [86,110,111,112]. Another study mentioned that during the administration of *A. sisalana* Perrine agro-waste on infested animals, no health issues were presented. Thus, its application was considered safe [113]. 

As it mentioned previously, leaf juice of *A. sisalana* has been the most studied agro-waste for its nematicide properties. Hecogenin, succinic acid, mannitol, homoisoflavonoids, and kaempferol were some of the metabolites identified in aqueous sisal liquid agro-waste in different research [114,115].

### 4.5. Antifungal Activity

The use of extracts from agave agro-wastes as bioweapons against phytopathogenic fungus is another potential approach in agricultural areas. In general, *Postia placenta*, *Alternaria brassicae*, *Fussarum oxysporum*, *P. aphanidermatum*, *Phytophthora cinnamomi*, *Penicillium digitatum*, *Sclerotium rolfsii*, *Pyricularia oryzae* are fungi agents that are causing different plant diseases that affect industrial crops. However, different *Agave* species were efficient in their biologic control.

Some *Agave* species such as *A. americana* L., *A. sisalana* Perrine, *A. scabra* Salm-Dyck, *A. marginata*, *A. ferox* K. Koch, *A. lechuguilla* Torr., *A. montana* Villareal and *A. sisalana* Perrine inhibited mycelial hyphal growth or showed antisporulant effects against the previous fungi [116,117,118,119,120,121]. The effective concentration, extract type, type of inhibition, and affected crop were summarized in Table 4. 

Maazoun, Hamdane, et al. [122] confirmed the antifungal effect of *A. americana* L. leaf extract against *P. digitatum*. Besides, the protease activity of this microorganism was inhibited at an IC_50_ concentration of 108.03 µg/mL. Another finding was that long exposition times of the extract to *P. digitatum* brought about the release of cell constituents which were correlated with the presence of saponins. On the other hand, the *A. americana* L. crude extract showed an antisporulant effect against *S. graminicola*. This effect was held after the extracts were diluted 10-fold. However, high diluted rates led to a lost antifungal effect [133].

The use of organic solvents such as methanol, n-hexane, and chloroform showed high antifungal activity [38,122]. Although, their application might be limited due to environmental issues. Castillo et al. [124] employed lanolin and cocoa butter instead of organic solvents on *A. lechuguilla* Torr. leaves with high tannin content. Additionally, these emulsions inhibited the mycelium growth of *R. solani* at concentrations of 1.70 × 10^4^ and 6.72 × 10^3^ mg/L, respectively. Similarly, Francisco et al. [119] confirmed the antifungal activity of lanolin extract of this species against *P. cinnamomi* at IC_50_ of 23.07 mg/mL. In contrast, lanolin extract exhibited an antifungal concentration lower than ethanolic extract but similarly to aqueous extract (28.87 mg/mL), which suggested that lanolin emulsions can also be efficient solvents to recover bioactive compounds. 

The regulation of fungal disease of downy mildew on *vitis vinifera L* through *A. cantala* (Haw.) Roxb. Ex Salm-Dyck leaf extracts was investigated by Vishal Naik et al. [132]. It found that ten days of after the extract was sprayed, it stimulated the enzymatic activity of polyphenol oxidase and catalase. Such enzymes are involved in the production of reactive oxygen species and quinones, which act as a defense response against phytopathogenic fungi. On the same topic, another study reported that the leaf extract of *A. cantala* (Haw.) Roxb. Ex Salm-Dyck contained high polyphenolic content with radical scavenger capacity [134]. Thus, by the previous study, the content of phenolic compounds might play a key role in the regulation of fungi diseases.

The chemical nature of the secondary metabolites was a factor that influenced antifungal activity. Yang et al. [135] isolated different saponin glycosides from *A. americana* leaves and found that tigogenin tetraglycosides with one xylose unit enhanced this effect while two or more xylose units decreased it. Therefore, the sugar chains impacted the antifungal potential of glycosylated steroidal saponins.

The agave crude extracts have also been referred to as excellent fungal toxin suppressors. Rosas-Taraco et al. [125] found that the aqueous extract of *A. americana* L. leaves decreased norsolorinic acid production by 75% at a concentration of 10 mg/mL. Norsolorinic acid is a precursor to the aflatoxin biosynthetic pathway. As a result of this, it reduced the aflatoxin concentration on *A. parasiticus*. Another study confirmed that aqueous extracts of *A. asperrima* Jacobi and *A. striata* Gentry inhibited 99% of the production of cyclopiazonic acid, a toxin biosynthesized by *A. flavus* on infested corn during storage [136]. Thus, agave extracts could have a useful application on cereal crop protection. 

### 4.6. Antiparasitic Activity

There is little information about the potential of agave agro-waste extracts against parasites that are harmful to human health. At least two studies informed the antiparasitic activity against *Leishmania donovani*, the causal agent of visceral leishmaniasis. 

According to the research of Singh et al. [137], the *A. americana* L. leaf extracts at 0.05 mg/L killed amastigotes and promastigotes of *L. donovani*, but this effect was preferably observed on amastigotes with 100% of mortality. Besides, the author compared the toxicity of extracts to amphotericin B, which resulted similarly to this commercial drug that is often used to treat visceral leishmaniasis. High costs, however, are a limitation in their treatment.

A second study reported that fractionated ethyl-acetate extracts from *A. americana* L. leaves obtained 100% mortality at 50 µg/mL against promastigotes and intracellular amastigotes of *L. donovani* at IC_50_ of 25 µg/mL. The application at effective doses increased nitric oxide levels in macrophages infested with amastigotes, which was suggested as a possible action mechanism of the extracts. However, after 48 h of dosing, a cytotoxic and hemolytic effect on macrophages was observed [138]. 

A third study reported by Quintanilla-Licea et al. [139] informed the antiparasitic activity of *A. lechuguilla* Torr. against *Entamoeba hystolytica*, the causal agent of amoebiasis, with a 69.66% growth inhibition; nevertheless, no median lethal dose was referred. More studies are required in this area, which offers a line of research to discover the antiparasitic effect of new molecules.

## 5. Conclusions

The hunting of novel bioactive molecules of the genus *Agave* and their biological activities shows that these plants are a valuable source of therapeutic agents, which can help generate new therapies for autoimmune, inflammatory, and chronic degenerative diseases. On the other hand, its use to obtain natural products might be a strategy to revalorize the agave agro-wastes, which could benefit the livestock and agricultural sectors due to the presence of secondary metabolites that play a protective role in bioweapons in pest control. Overall, natural extracts from agave agro-wastes might be an alternative for agricultural purposes because of their safety and low toxicity to the non-target organism compared to synthetic pesticides, which are harmful to the environment and human health. Being a material rich in different compounds of agro-industrial interest, future research focused on the isolation, purification, and protection of the secondary metabolites of the agave using environmentally friendly processes, as well as the latest sensitive and reliable technology instrumentation for their identification remain topics of interest for the use of agave agro-waste. On the other hand, delving into the development of products based on the use of these pure metabolites or its extracts, the evaluation of its stability and bioactivity, as well as its application in different areas of science will allow us to contribute to the circular economy of the production chain of the Agave.

## Figures and Tables

**Table 1:** Medicinal uses of the *Agave* genus.

Species/Tissue	Medicinal Use	References
*A. potatorum* Zucc./leaf	Healing and cicatrization of wounds	[18]
	Infusions to heal respiratory infections	
*A. americana* L./leaf, roots	Healing and cicatrization of wounds from leaf gel	[19]
	Syphilis treatment, anesthetic, headache, rheumatic pain, and broken bones	[15]
	Treatment of gout disease, infections, and burns	[20]
*A. sisalana/leaf*	Treatment for tick-borne diseases	[14]
	Stomach detoxifier and constipation	[21]
	Antimicrobial against pathogen biota of the intestines and stomach	[22]
*A. sisalana/roots*	Antidiarrheic	[23]
*A. sisalana/leaf* *A. karattos* Linnaeous/*leaf*	Treatment against meningitis and sciatica	[16]
*A. angustifolia* Haw./*leaf, roots, fibers, leaf juice and root*	Skin eruptions, kidney disease, and hepatic affections. It has antiparasitic and anti-hemorrhagic qualities	[17]

**Table 2:** Biological activity of identified/isolated phytochemicals from the *Agave* genus.

Specie/Tissue	Compound(s)	Biological Activity	Dose	Study Model	References
		*Phenolic Compounds*			
*A. durangensis* Gentry Leaf	Flavonoid glycosides, aglycones, phenolic acids, proanthocyanins	Stimulant expression of enzymes	-	*N. crassa* ITD-G7 *A. niger* ITD-G1	[43]
*A. lechuguilla* Torr. Leaf	Afzlequin-4-β-quercetin; dimeric flavonoids, quercetin, kaempferol	Anticancer	IC_50_: 6.96 µg/mL	SK-LU-1 cells	[47]
*A. americana* L. Leaf	Flavonoid glycosides of kaempferol, quercetin, isorhamnetin, ellagic acid hexoside	Insecticide Anti-repellent	10.55 µg/insect RD: 0.055 µg/cm^2^	*Sytophilus oryzae*	[49]
*A. lechuguilla* Leaf	2,4,6-trinitrophnol, flavonoids, carboxylic acids, 2-aminomethil-propanol	Insecticide	LD_50_: 1035 mg/mL	*Bemisia tabaci*	[50]
*A. pygmaea* Gentry *A. angustifolia* Haw. cv. *marginata* Leaf	Phenolic acids, flavonoid glycosides, homoisoflavonoids	Anti-inflammatory Anti-ulcerogenic	200 mg/kg 20 mg/kg	Wistar rats: rat paw stomach	[51]
*A. americana* L. Leaf	Apigenin	Antifungal	IC_50_: 72.15; 18.83 µM	*A. oryzae* α-amylase	[52]
*A. americana* L. Leaf	p-coumaric acid, puerarin	Antidiabetic	IC_50_: 98.8 µM, 3.87 µM	Human α-amylase	[53]
*A. sisalana* Fiber	Acylated glycosyl flavonoids, flavonoid glycosides, phenolic acids	Stimulant and growth protection of microgreens	-	Microgreens	[54]
***Saponins***					
*A. salmiana* Sap	Kammogenin-pentaglucoside and tetraglycoside, Gentrogenin pentaglucoside	Anticancer	IC_50_ 82.7, 108.4 µg/mL	HT-29 cells	[55]
*A. americana* L. *marginata* Hort. Leaf	Cantala-saponin-1	Anti-neuroinflammatory	5-10 mg/kg	Rat brain	[56]
*A. salmiana* Sap	Kammogenin-glycosides	Anticancer	50 µg/mL	Hep-G2, CaCo-2 cells	[57]
*A. marmorata* Roezl. Leaf	Smilagenin-diglycoside	Immunomodulatory Anti-inflammatory	IC_50_: 50 mg/mL, 0.086 mg/mL	RAW 264.7 cells	[58]
*A. sisalana* Perrine Leaf juice	Glycosylated steroidal saponins Hecogenin-pentaglycoside	Cytotoxic	IC_50_: 125 µg/mL	C6 cells	[59]
*A. sisalana* Perrine Leaf juice	Steroidal saponins	Antineoplastic	50 µg/mL	Vero-cells Human lymphocytes, rats	[60]
*A. angustifolia* var. *marginata* Leaf	Steroidal-hexaglycosylated saponin	Anti-ulcerogenic	100 mg/kg	Rat stomach	[61]
*A. seemanniana* Jacobi Leaf	Steroidal saponins	Analgesic Anti-inflammatory Anti-ulcerogenic	100 mg/kg	Rat stomach	[62]
***Phytosterols***					
*A. tequilana* Weber Piña	β-sitosterol-glycoside, stigmasta-3,5-dien-7-one Cicloartenone, cycloartenol	Immunomodulatory Anti-inflammatory	50 mg/kg	Urine, blood serum, kidney rat	[63]
*A. angustifolia* Haw. Leaf	3-*O*-[(6′-*O*-palmitoyl)-β-d-glucopiranosyl-sitosterol]	Immunomodulatory Anti-inflammatory	50 mg/kg	Mice ear edema	[64]

RD: repellent dose, IC_50_: Half maximal inhibitory concentration, LD_50_: Median lethal dose.

**Table 3:** Biopesticide potential of agave extracts.

Species/Tissue	Extract	Targeted Organism	Affected Organism	Biological Activity	LD_50_ (mg/mL)	MI (%)	t	References
*A. americana* L. Leaf	M	*Sitophilus oryzae* (L.)	*Triticum durum* Desf.	Insecticide Repellent	8.99 µg/cm^2^ 0.055 µg/cm^2^	50	24 h	[49]
*A. lechuguilla* Torr. Leaf	HA	*Bemisia tabaci*	*Phaseolus vulgaris*	Insecticide	1035 mg/L	-	72 h	[50]
*A. sisalana* Perrine Leaf	HA	*Dactylopius opuntiae*	*Opuntia ficus-indica*	Insecticide	17, 46	51, 97	10 Days	[77]
*A. sisalana* Perrine Leaf juice	A	*Aedes aegyptii*	*-*	Ovicide Cytotoxic	6	73.8	24 h	[78]
*A. sisalana* Perrine Leaf	Hxn	*Callosobruchus maculatus*	*Phaseolus* spp.	Insecticide Ovicide	100	100	24–96 h	[80]
*A. americana* var. *Marginata* Trel. Leaf	A	*Brevicoryne brassicae*	*Brassica oleracea* L. var. *acephala*	Insecticide	0.750	>70	3 h	[81]
*A. americana* Leaf	A	*Aphis gossypii*	*Gossypium hirsitum* L.	Insecticide	25, 50	70.3, 92.5	24, 36 h	[82]
*A. americana* L. Leaf	A	*Olygonichus ilicis*	*Coffea arabica* L. *Coffea canephora*	Insecticide	4% (*v*/*v*)	100	72 h	[84]
*A. angustifolia* Haw. Leaf	A	*Tetranychus urticae*	*Fragaria* L. *Fragaria*	Insecticide	10% (*v*/*v*)	88	5 Days	[85]
*A. tequilana* Weber Leaf juice	Hxn	*Bemisia tabaci*	*Solanum lycopersicum*	Insecticide	20–40	90	24 h	[86]
*A. sisalana* Perrine Leaf juice	A	*Tetranycuhs urticae*	*Gossypium hirsitum* L.	Insecticide	-	100	35–65 Days	[87]
*A. attenuata* Leaf	A	*Dyuraphis noxia*	*Triticum* spp.	Insecticide	0.838	98.75	24 h	[88]
*A. filifera* *Whole plant*	A	*Biomphalaria alexandrina*	Humans	Molluscicide Ovicide	42.30 85.62	40.5	6 weeks	[89]
*A. celsii* Leaf	M	*Biomphalaria alexandrina*	Humans	Molluscicide	40, 73	-	24 h	[90]
*A. lophantha* Leaf	B	*Biomphalaria alexandrina*	Humans	Molluscicide	17, 100	100	48 h	[91]
*A. attenuata* Leaf	A	*Bulinus africanus*	Humans	Molluscicide	65	-	24 h	[92]
*A. legrelliana* Jacobi, *A. americana marginata* L. Leaf juice	A	*Praticolella griseola* (Pfeiffer)	Humans Animals Crops	Molluscicide	50% mL/L	82.5 77.5	7 Days	[93]
*A. americana* var. *expansa* Leaf	A	*Melanoides tuberculata* (Thiaridae)	Native mollusks Humans	Molluscicide	1.01 mL/L	65	24 h	[94]
*A. americana* Leaf	B	*Indoplanorbis exustus* *Lymnaea luteola* *Gyraulus convexiusculus*	Humans Animals	Molluscicide Ovicide	21.9, 18.5, 16 16.4, 15.2, 7.5	-	24 h	[95]
*A. sisalana* Perrine Leaf	A, B, E	*Pomacea canaliculata* L.	*Aegle marmelos*	Molluscicide	35.3 g/L, 93.3, 298.6	-	72 h	[96]
*A. sisalana* *A. attenuata* Leaf	E	*Biomphalaria alexandrina*	Humans	Molluscicide	82, 101	-	72 h	[97]
*A. sisalana* Perrine Leaf juice	A	*A. aegypti*	Humans	Larvicide	4.5, 6.5	100	12, 24 h	[98]
*A. sisalana* Perrine Leaf	A, M	*A. aegypti* *C. quinquefasciatus* *A. stephensi*	Humans	Larvicide	86, 76, 75 82, 220, 36	100	24 h	[99]
*A. sisalana* Leaf	A	*A. aegypti* *C. quinquefasciatus*	Humans	Larvicide	100	100	3–4 Days	[100]
*A. cantala* (Haw.) Roxb. Ex Salm-Dyck Leaf	Acn	*Plutella xylostella* (L.)	*Brassica oleracea*	Larvicide	60	35.77	12 h	[101]
*A. sisalana* Perrine Leaf juice	A	*Radopholus similis*	*Musa x paradisiaca*	Nematicide	5, 25% (*v*/*v*)	90.1, 99.2	24, 48 h	[102]
*A. sislana* Perrine Leaf juice	A	*Meloidogyne javanica*	*Solanum lycopersicum*	Nematicide	20% (*v*/*v*)	100	48 h	[103]
*A. sisalana* Perrine Leaf juice	A	*Scutellomena bradys*	*Dioscorea esculenta*	Nematicide	20–40% (*v*/*v*)	100		[104]
*A. angustifolia* Haw. Leaf	Acn	*Sitophilus zeamais* Motschulsky	*Zea mays* L.	Insecticide	5% (*v*/*v*)	34	72 h	[105]
*A. angustifolia marginata* Leaf	A	*A. aegypti* *C. quinquefasciatus* *A. stephensi*	Humans	Larvicide	28.27 µg/mL 100 µg/mL	100	12 h	[106]

HA: Hydroalcoholic, A: Aqueous, Hxn: n-Hexane, M: Methanolic, E: Ethanolic, Acn: Acetone, B: Butanol, MI %: Mortality index, DL_50_: Median lethal dose, t: time.

**Table 4:** Antifungal potential of agave extracts.

Species/Tissue	Extract	Target Organism	Affected Crop	Biological Activity	LD_50_/MIC	IG (%)	t	References
*A. montana* Villareal *A. marginata* *A. americana* L. *A. ferox* K. Koch Leaf	AM	*Postia placenta*	*-*	Antifungal	-	69.31 ^b^	7 days	[116]
*A. americana* L. Leaf	M	*Alternaria brassicae*	*Brassica juncea*	Antifungal	40 µg/mL	-	4 days	[117]
*A. sisalana* Perrine Leaf	A	*Pyricularia oryzae*	*Oryza sativa* L.	Antifungal	3% (*v*/*v*)	100 ^a^	10 days	[118]
*A. lechuguilla* Torr. Leaf	A L	*Phytophthora cinnamomi*	*Persea americana Mill.*	Antifungal	28.87 23.07 mg/mL	60 ^a^	-	[119]
*A. americana* L. Leaf	A	*Fusarium oxysporum* *Pythium aphanidermatum*	*Zingiber officinale* Rosc.	Antifungal	-	67.98 ^a^ 76.75 ^a^	96 h	[120]
*A. scabra, Salm Dyck* Leaf	HA	*Botrytis cinerea* *Penicillium* sp.	*-*	Antifungal	-	90 ^c^ 86.6 ^c^	7 days	[121]
*A. americana* L. Leaf	M	*Penicillium digitatum* *Sclerotium rolfsii*	*-*	Antifungal	11.86 21.97 µg/well	87.73 ^a^ 80.81 ^a^	9 days	[122]
*A. americana* L. Leaf	M	*Sclerospora graminicola*	*Cenchrus americanus*	Antisporulant	-	-	12–14 h	[123]
*A. lechuguilla* Torr. Leaf	L C	*Rhizoctonia solani*	*-*	Antifungal	1.70 × 10^4 a^ 6.72 × 10^3 a^ mg/L	14.1 ^c^	-	[124]
*A. americana* L. Leaf	A	*Aspergillus parasiticus*	*Cereal crops*	Reduce aflatoxin production	-	-	7 days	[125]
- Leaf	E	*Penicillium* sp. *Penicillium variable* *Fusarium verticillioides*	*Phaseolus lunatus* L.	Antifungal	-	85 ^c^ 92.5 ^c^ 100 ^c^	7 days	[126]
*A. americana* L. Leaf	A	*Phytophthora megakarya*	*Theobroma cacao* L.	Antifungal	60 mg/mL	54.33 ^a^	7 days	[127]
*A. sisalana* Perrine Leaf juice	A	*Lasiodiplodia theobromae*	*-*	Antifungal		75.60 ^a^	3 days	[128]
*A. americana* L. Leaf	-	*Leveillula taurica* (Lev.) Arn	*Capsicum annum* L.	Weak antifungal	-	-	10 days	[129]
*A. cantala* (Haw.) Roxb. Ex Salm-Dyck Leaf	A	*Peronospora farinosa*	*Vinis vinifera* L.	Fungal protection	-	-	10 days	[130]
*A. marginata* *A. americana* L. *A. ferox* K. Koch Leaf	AM	*Macrophomina phaseolina*	*-*	Antifungal	-	64.75 ^b^ 61.69 ^b^ 59.39 ^b^	7 days	[131]
*A. cantala* (Haw.) Roxb. Ex Salm-Dyck Leaf	A	*Plasmopara viticola*	*Vinis vinifera* L.	Fungal protection	-	-	10 days	[132]
*A. americana* L. Leaf	A	*Sclerospora graminicola*	*Cenchrus americanus*	Antisporulant	-	-	12–14 h	[133]

LD_50_: Median lethal dose, % IG: Inhibition growth percentage, MIC: Minimal inhibitory concentration, E: Ethanolic, M: Methanol, A: Aqueous, L: Lanolin, HA: Hydroalcoholic, AM: Aqueous methanol, C: Cocoa butter, ^a^: Mycelial growth inhibition, ^b^: Hyphal growth inhibition, ^c^: Growth inhibition.

## Data Availability

Not applicable.

## Sample Availability

Samples of the compounds are not available from the authors.
